# Heuristics and biases: interactions among numeracy, ability, and reflectiveness predict normative responding

**DOI:** 10.3389/fpsyg.2014.00665

**Published:** 2014-07-02

**Authors:** Paul A. Klaczynski

**Affiliations:** Decision Making and Development, School of Psychological Science, University of Northern ColoradoGreeley, CO, USA

**Keywords:** normative, heuristics and biases, analytic processing, moderator effects, numeracy

## Abstract

In Stanovich's (2009a, 2011) dual-process theory, analytic processing occurs in the algorithmic and reflective minds. Thinking dispositions, indexes of reflective mind functioning, are believed to regulate operations at the algorithmic level, indexed by general cognitive ability. General limitations at the algorithmic level impose constraints on, and affect the adequacy of, specific strategies and abilities (e.g., numeracy). In a study of 216 undergraduates, the hypothesis that thinking dispositions and general ability moderate the relationship between numeracy (understanding of mathematical concepts and attention to numerical information) and normative responses on probabilistic heuristics and biases (HB) problems was tested. Although all three individual difference measures predicted normative responses, the numeracy-normative response association depended on thinking dispositions and general ability. Specifically, numeracy directly affected normative responding only at relatively high levels of thinking dispositions and general ability. At low levels of thinking dispositions, neither general ability nor numeric skills related to normative responses. Discussion focuses on the consistency of these findings with the hypothesis that the implementation of specific skills is constrained by limitations at both the reflective level and the algorithmic level, methodological limitations that prohibit definitive conclusions, and alternative explanations.

## Introduction

When the standards against which they are evaluated are traditional norms, performance on heuristics and biases (HB) tasks is often poor (Kahneman et al., 1982; Reyna and Brainerd, 1995; Stanovich, 1999). Underlying most views of the “normative/descriptive gap” (see Baron, 2008) is the assumption that rational thinking is “bounded” by information processing limitations (e.g., working memory, processing speed). In accord with this view, measured intelligence, generally assumed to index these processing limitations, relates positively to normative responses on several HB tasks. To the extent that measured intelligence accurately taps individual differences in cognitive capacity, these findings partially support the “bounded rationality” hypothesis. The general modesty of the correlations (*r*s range = 0.20–0.45; see Stanovich and West, 2008) implies, however, that considerable variance in responding cannot be easily attributed to computational limitations (see also Reyna, 2000).

Evidence that differences in general ability account for 20% (or less) of the variability in normative responses was at least partially responsible for research on the associations between responses and less “bounded” individual difference variables. Thus, in addition to research on specific intellectual competencies (e.g., inhibition; Markovits et al., 2009; De Neys, 2012; Markovits, 2013), the focus of numerous investigations has been the relationship between thinking dispositions (TD) and HB responses (e.g., Stanovich and West, 1998; see Stanovich, 2009b, 2012). Thinking dispositions—relatively malleable cognitive styles, beliefs, intellectual values, and motivations to manage cognitive resources (e.g., expending effort, guarding against impulsivity, valuing deliberate thinking, openness to using different strategies)—often account for variance in performance independently from general ability (Stanovich and West, 1998, 2000; Klaczynski and Lavallee, 2005; West et al., 2008; Toplak et al., 2011).

Research on TD and general ability (GA) has led to theoretical models that distinguish between two levels of analytic processing. The most common distinction in dual-process theories is between autonomous (or “Type I”) processing and analytic (or “Type II”) processing (e.g., Evans, 2009, 2011; Klaczynski, 2009; Barrouillet, 2011; Stanovich, 2011; Evans and Stanovich, 2013). Autonomous processing is triggered by task/situational factors, operates without conscious awareness and automatically activates situationally-relevant heuristics and other memories (e.g., procedural) that can serve as the basis for inferences and judgments. Analytic processing is conscious, deliberate, and cognitively demanding and is responsible for judging the adequacy of autonomously-produced representations and responses, determining whether to override autonomous processing, and engaging conscious reasoning and decision making abilities (see Stanovich, 1999, 2009a; Klaczynski, 2004; Evans, 2007). When predominant, analytic processing guides the selection and operation of the cognitive strategies and underlies complex reasoning and computations (Stanovich, 2011).

Stanovich (2004, 2009a; Stanovich and West, 2008; Stanovich et al., 2011) has proposed the analytic processes are best conceived as operating in two related “minds”: The reflective mind and the algorithmic mind—hereafter referred to as the reflective and algorithmic levels. Reflective-level operations, generally indexed by measures of epistemic understanding and thinking dispositions, regulate or govern algorithmic-level activities and are therefore metacognitive in nature. The algorithmic level, most often indexed by measures of intelligence, comprises general cognitive competencies, information processing efficiency (e.g., working memory), reasoning abilities (inductive, deductive), and specific computational and logical rules, strategies, and abilities. This description suggests that the algorithmic level can be partitioned into (a) general abilities, resources, and limitations on processing efficiency and (b) specific abilities or “micro-strategies” (see Stanovich, 2009a, p. 71). General processing resources are superordinate to specific abilities in the sense that, in the absence of sufficient resources, even individuals who possess the abilities (e.g., numeracy, described subsequently) to solve particular problems will be incapable of fully utilizing those abilities and will therefore err in their attempts.

The conceptual relationships among the reflective level, general algorithmic-level resources, and specific algorithmic abilities can be summarized as follows. First, because the reflective level guides operations (e.g., specific strategy selection, computation monitoring, response evaluation) at the algorithmic level, it is superordinate to both general algorithmic resources and specific algorithmic skills. Second, despite being “subordinate,” available algorithmic resources necessarily limit the efficiency of reflective-level functions. Third, the same algorithmic limitations impose constraints on the quality (e.g., complexity) and functionality of specific skills.

The present research was intended to provide a preliminary test of the model of analytic processing outlined above and examine the associations among thinking dispositions, general ability, and numeracy. Broadly defined, numeracy is set of specific algorithmic “micro-strategies” encompassing individuals' understanding of, and ability to assign meaning to, mathematical concepts (Nelson et al., 2008; Peters, 2012). Because numerous HB tasks require at least a minimal understanding of probabilities, numeracy is an algorithmic skill set with considerable promise for advancing our understanding of the processes underlying performance. Indeed, extant research indicates that numeracy is associated with general ability and explains variance on some HB tasks beyond that attributable to general ability and more specific aspects of algorithmic competence (e.g., inhibition; Peters et al., 2006; Nelson et al., 2008; Liberali et al., 2011; Toplak et al., 2011). Despite these findings, several hypotheses directly relevant to Stanovich's theory of analytic processing have not been examined.

**Figure 1:**
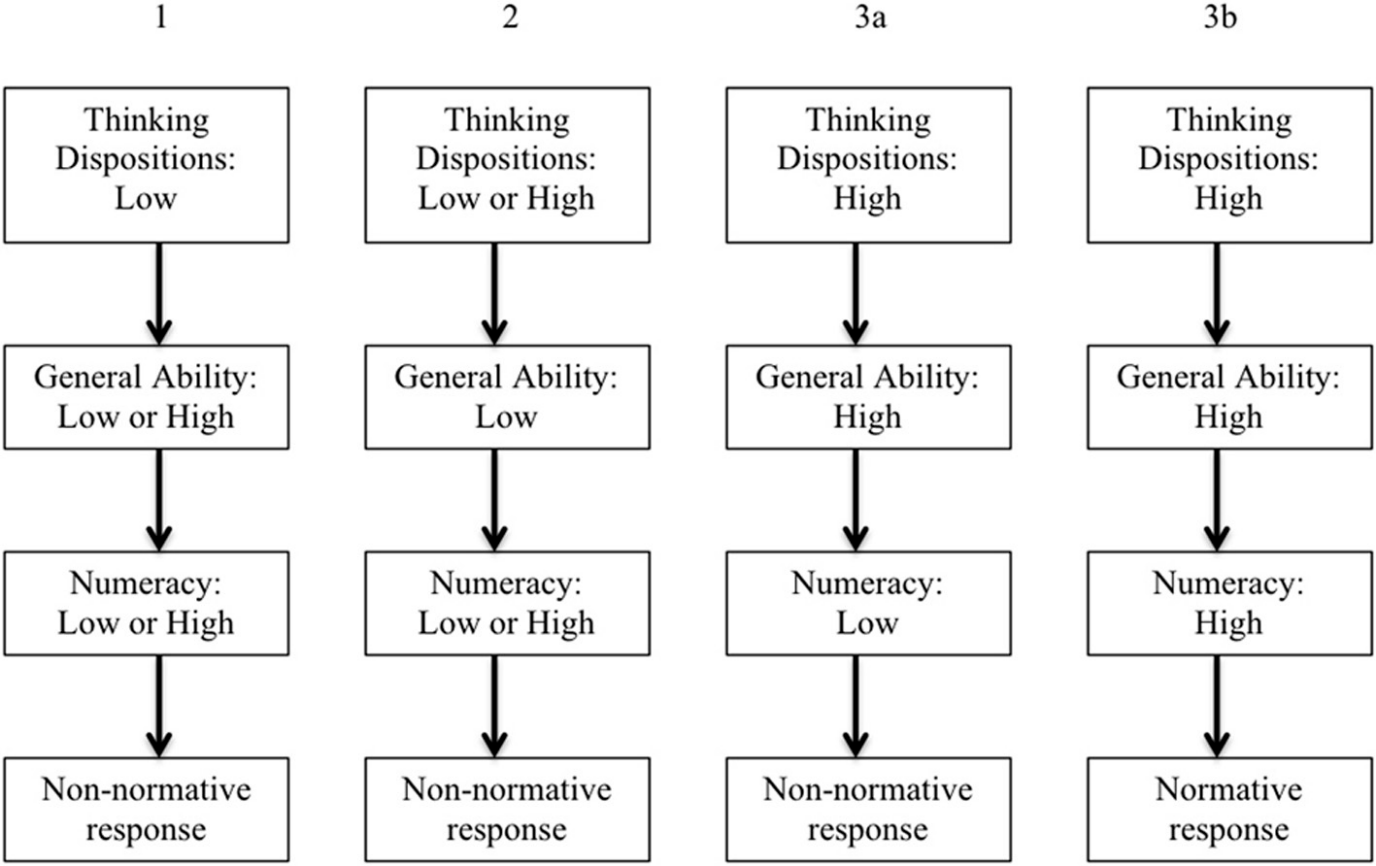
**Predicted relationships among thinking dispositions, general ability, numeracy, and normative responses on probabilistic HB tasks**.

Specifically, the view of Stanovich's theory espoused here, that reflective operations guide general algorithmic operations and that both reflective and algorithmic operations are important determinants of whether numeric skills are used to generate normative responses, implies specific conditions under which numeracy predicts normative responses on probabilistic tasks. Because reflective operations are critical to judging the adequacy of automatically-activated representations and responses, determining whether decoupling is necessary, understanding task requirements (e.g., whether problems require numeric computations), selecting specific algorithmic skills, monitoring computational operations, and evaluating outcomes, a first condition is adequate reflective-level functioning. A second necessary condition is the availability of sufficient general algorithmic capacity: Algorithmic resources (e.g., working memory) are required not only to perform reflective operations and sustain decoupled representations but also to effectively utilize numeric abilities and conduct computations (Stanovich and West, 2008). Thus, the effects of numeracy on responses should depend on (i.e., be moderated by) thinking dispositions and cognitive ability. This conjecture led to the hypotheses described below and depicted in Figure 1.

1. *Inadequate reflective-level regulation*. Inadequacies at the reflective level should result in poor management of general algorithmic resources, little attention to representation quality or consideration of alternative representations, errors in specific ability selection, and little monitoring of algorithmic operations. Therefore, regardless of general ability, numeracy was not expected to relate to normative responding among participants with poorly developed thinking dispositions.
2. *Inadequate general algorithmic resources*. Because algorithmic resources limit the efficiency of both reflective-level functions and numeric operations, participants low in general ability were expected to respond non-normatively—regardless of thinking dispositions and numeric ability.
3. *Low numeric ability*. Regardless of levels of reflective and algorithmic functioning, to perform well on probabilistic problems, individuals must have adequate numeric abilities. Those with poor numeric abilities were expected to respond non-normatively—regardless of reflective skills (TD) and algorithmic resources (GA).
4. *High numeric ability.* From the model described previously and the preceding hypotheses, it follows that, among participants high in numeric ability, only those who also have high levels of thinking dispositions *and* general intellectual ability would respond normatively^1^.

The above predictions apply only to conflict problems—that is, problems wherein different responses are implied by task content (e.g., stereotype-activating information) and task structure (e.g., probability information). In contrast to *conflict* (i.e., CN) problems, on *no-conflict* (i.e., N-CN) problems, responses triggered automatically by task content are the same (i.e., normative) as responses based correct application of analytic abilities (De Neys, 2012). Although responses on N-CN problems have been examined in some investigations (e.g., De Neys and Van Gelder, 2009; Thompson and Johnson, 2014; see also research on belief-biased reasoning; e.g., Evans et al., 1983), N-CN problems are often not examined in HB research. However, because normative responses should be considerably more frequent on N-CN problems than on CN problems and because N-CN responses should not be diagnostic of underlying processes, performance on N-CN problems should correlate with neither performance on CN problems nor the individual difference measures. Preliminary analyses were intended to explore these hypotheses for no-conflict problems (in a sense, the N-CN problems served as control problems; see De Neys, 2012).

## Methods

### Participants

As part of a larger investigation, 219 undergraduates earned course credit for participating in single 60–80 min session (in groups of 4–8 students) during which they reported their verbal and quantitative SAT scores, completed measures of numeracy, general ability, and thinking dispositions, and responded to a battery of HB tasks.

### Materials

#### Thinking dispositions

The 52-item TD questionnaire, based on similar measures used by Stanovich and West (e.g., Stanovich and West, 1998, 2007) and Klaczynski (e.g., Klaczynski and Lavallee, 2005), contained five subscales (items were intermixed randomly). The 10-item *flexible thinking* scale measured willingness to take into account multiple perspectives and beliefs that complex decisions cannot be reduced to “either-or” choices (Macpherson and Stanovich, 2007). The 10-item *reflectiveness* vs. *intuition* scale assessed beliefs that logic and careful analysis leads to better decisions than reliance on intuitions (Epstein et al., 1995). The 12-item *need for cognition* scale measured valuation of intellectual challenges, complex thinking, and logical deliberation (see Cacioppo et al., 1996). The 14-item *impulsive decision making* scale tapped tendencies to make decisions “on the spur of the moment” (i.e., without considering consequences or alternatives) and believe that the best decisions are made quickly (see Patton et al., 1995). The 8-item *epistemic regulation* scale indexed understanding that belief conflicts can be resolved by considering the best available evidence (based on Kuhn, 2006 and Moshman, 2013). Participants responded to each item on a 6-point scale (1 = *strongly disagree*; 6 = *strongly agree*).

To reduce the number of analyses, a composite TD score was computed (*M* = 161.68, *SD* = 13.85). The composite was justified by the positive correlations among subscales (smallest *r* = 0.25) and the higher internal consistency (α = 0.78) and stronger correlations with responses for the composite than for the subscales.

#### General ability

Both verbal ability and inductive reasoning ability were assessed. Verbal ability, best indexed by vocabulary, is among the foremost indicators of global and crystallized intelligence. Fluid intelligence, perhaps the best indicator of algorithmic-level functioning (Stanovich, 2009a,b), was indexed by scores on an inductive reasoning test.

***Verbal ability***. A 30-item vocabulary test (*M* = 21.87; *SD* = 2.72), based on the Shipley-2 vocabulary test (Shipley et al., 2010), was administered. Pilot testing indicated a correlation of 0.89 between the revised and the original tests. The Shipley-2 has excellent internal and test-retest reliability and relates moderately/strongly to academic achievement, general intelligence, and other indexes of crystallized intelligence (Prokosch et al., 2005; Kaya et al., 2012). On each item, a target word (e.g., jocose) was followed by four options (e.g., humorous, paltry, fervid, plain). Correct responses required selecting the word with same meaning as the target. Three minutes were given to complete as many items as possible.

***Inductive ability***. A 20-item inductive reasoning test (*M* = 10.75; *SD* = 1.72) was administered. Items were selected after removing the easiest and most difficult items from the PMA Letter Sets test (Thurstone, 1962). In pilot testing, the original PMA and the reduced version were correlated highly (*r* = 0.84). Scores on the original test and shortened versions of the test correlate well with general intelligence and other indexes of fluid intelligence (Hertzog and Bleckley, 2001; Colom et al., 2007). From five sets of four letters (e.g., ACDE, MOPQ, FGIJ, DFGH, TVWX), participants indicated the set that did not belong with the other sets (e.g., FGIJ) and completed as many items as they could in 12 min.

A composite ability score was analyzed for several reasons. First, inductive and verbal scores correlated moderately (*r* = 0.47; Kaya et al., 2012, reported a similar correlation). Second, scores on the two measures related similarly to normative responses. Third, the combined ability score correlated better (see **Table 2**) with normative responses than inductive ability (*r*s ranged from 0.21 to 0.28) or verbal ability (*r*s ranged from 0.22 to 0.27).

#### Numeracy

Participants completed a 20-item objective numeracy test (α = 0.82; *M* = 11.39, *SD* = 3.53). Objective numeracy tests (in contrast to subjective tests) contain items that measure basic probability skills, such as those involved in converting ratios to percentages (and vice versa) and analyzing fractions (e.g., 2/20 vs. 3/40) to determine relative probabilities. The numeracy test (available from the author) was similar to the tests used by Peters et al. (2006), Nelson et al. (2008), and Liberali et al. (2011) and an included items from (or adapted from) Lipkus et al. (2001), Garfield (2003), Irwin and Irwin (2005), and Klaczynski and Amsel (2014).

Each item included a problem that required understanding a probabilistic concept and selecting, from 3–5 response options, the correct solution (e.g., from a list of 20 names, the chances a randomly selected name would begin an “A”; the probability that a randomly selected person would be a doctor who also enjoys hiking in a group of 100 people with three doctors and eight people who enjoy hiking). The predictive value and validity of the test were established in two developmental studies of responses on HB problems similar to those described subsequently. In both studies, numeracy increased with age and accounted for more variance in normative responding than age or ability. Using a similar measure, Klaczynski and Amsel (2014) found that numeracy predicted differences on probabilistic reasoning tasks better than age or nationality (Chinese or American).

#### Heuristics and biases tasks

Given the definition of numeracy given previously, numeracy should be a better predictor of normative responses on *probabilistic* HB problems than of normative responses on non-probabilistic problems. The battery, presented in one of four randomly determined orders and mixed with problems from a larger study (order was not related to responses on any HB task or to any of the individual difference measures), included eight base rate neglect (BR), eight law of large numbers (LLN), eight ratio bias (RB), and eight covariation judgment (COV) problems. For each of task (i.e., BR, LLN, RB, COV), there were four conflict (CN) problems and four no-conflict (N-CN) problems. On both the conflict and no-conflict versions of each task, normative scores could range from 0 to 4; mean proportions of normative responses are presented in the Results to increase the ease of comparing the findings with other research. Examples of conflict and no-conflict versions of each task are presented in the Supplementary material^2^.

***Base rate neglect problems***. Each problem intended to elicit base rate neglect contained two types of information: (1) Base rate data indicating the number of people in each of two groups and (2) descriptions of individual “targets” that were consistent with stereotypes associated with one group (e.g., knitting, gardening). On CN problems, target descriptions “pulled” for responses based on group stereotypes and the base rates (e.g., 125 17-year-olds and 25 50-year-olds) pulled for the normative response that targets were not likely to be members of the stereotyped groups. The stereotypes thus cued responses that conflicted with normative responses. The target descriptions in the N-CN problems were identical to those in the CN problems; however, on the N-CN problems the base rates (e.g., 25 17-year-olds and 125 50-year-olds) indicated that targets were likely in the stereotyped group. Normative responses were thus cued by both the stereotypes and the base rates (see also De Neys and Glumicic, 2008).

On each problem, participants judged target group membership on 4-point scales (e.g., 1 = *Very likely to be 17 years old*; 2 = *Somewhat likely 17 years old*; 3 = *Somewhat likely to be 50 years old;* 4 = *Very likely to be 50 years old*; reversed for half the problems). Consistent with previous studies (e.g., Toplak et al., 2014), responses on the CN problems were considered normative (scored “1”) when participants rated that targets as unlikely or very unlikely to be in the stereotyped group and responses on the N-CN problems were scored normative when participants rated targets as likely or very likely to be in the stereotyped group.

***Law of large numbers***. Adapted from Fong et al. (1986), Stanovich and West (1998), and Klaczynski (2001), these problems involved making decisions after reviewing arguments founded on large evidential samples and arguments based on small samples of personal and relatively vivid evidence. On CN problems, large sample arguments supported one decision and small sample arguments supported a different decision. On the N-CN problems, the large sample and small sample arguments supported the same decision. On four problems (two CN, two N-CN), the large sample arguments were presented before the small sample arguments. On the other four problems (two CN, two N-CN), the small sample arguments were presented first.

Participants indicated the decision they judged best on 4-point scales (1 = “Decision ‘A’ is a much better decision”; 2 = “Decision ‘A’ is a better decision”; 3 = “Decision ‘B’ is a better decision”; 4 = “Decision B is a much better decision,” where “Decision B” indicated preference for the large sample argument). For half the problems, the rating scale was reversed and later recoded; consequently, on both the CN and N-CN problems, ratings of 3 and 4 reflected greater reliance on the large sample arguments. Following Stanovich and West (1998), Klaczynski (2001), and Toplak et al. (2007), ratings ≥3 were considered normative and assigned scores of 1.

***Ratio bias***. On the RB problems (Denes-Raj and Epstein, 1994), participants judged whether targets (e.g., winning lottery tickets) were more likely if a person selected from a relatively large numerator/large denominator sample (e.g., nine winning tickets in 100 total tickets) or a relatively small numerator/small denominator sample (e.g., one winning ticket in 10 total tickets). The RB effect occurs when individuals believe that targets are more likely from relatively large samples than from relatively small samples. Reyna and Brainerd (2008) distinguished between heuristic RB problems (i.e., identical probabilities in the two samples) and non-optimal RB problems (i.e., probabilities favor the smaller sample). Although the RB effect has been reported on both heuristic and non-optimal problems, non-optimal problems were used in the present research because the normative response (e.g., on CN problems, targets were more likely from the smaller sample) was more similar to normative responses on the other tasks than was the normative response on heuristic problems (i.e., neither sample is more likely to yield a target).

On each problem, the absolute number of targets (i.e., numerators) and the total (i.e., targets plus non-targets; denominators) was higher in the large sample than in the small sample. On CN problems, target probability was higher in the smaller sample. By contrast, on N-CN problems, the absolute numbers of targets and the probabilities of targets were higher in the larger samples: Similar to the N-CN contingency detection problems (described next), normative selections could be based on calculating and comparing ratios or simply comparing numerators. On two CN and two N-CN problems, the small sample response was presented before by the large sample response; on the other CN and N-CN problems, the larger sample option was presented before the small sample option. A third option (that target probability was the same in the two samples) was always presented last. Participants judged which, if either, sample was more likely to yield a target (e.g., winning lottery ticket). Judgments were normative (scored “1”) when the small sample was selected on the CN problems and the large sample was selected on the N-CN problems.

***Covariation judgment problems***. Based on Wasserman et al. (1990) and modeled after the problems in Stanovich and West (1998), Klaczynski (2001), and De Neys and Van Gelder (2009), each problem described a hypothetical investigation of a potentially causal relationship between two variables. Descriptions were accompanied by 2 × 2 contingency tables summarizing the results (i.e., numbers of cases) in each of the four cells: (putative) cause-present/effect-present, cause-absent/effect-present, cause-absent/effect-present, and cause-absent/effect-absent (labeled the A–D cells; Wasserman et al., 1990). Relationship strength can be determined by computing phi (φ) or comparing conditional probabilities [A/(A + B) − C/(C + D)], although less precise ratio comparisons yield relationships in the same direction as φ. When Cell A is clearly larger and more salient than Cell B (and Cell C), adults often adopt the simple strategy of comparing numbers of cases in Cell A with the numbers of Cell B (or Cell C; see Alloy and Tabachnik, 1984; Maldonado et al., 2006). As discussed by fuzzy-trace theorists, this numerosity bias is similar to that found on RB problems (see Reyna and Brainerd, 2008).

On the CN problems, the absolute numbers in Cell A (e.g., 35) were greater than the numbers in cell B (e.g., 26) and cell C (e.g., 27), but the φ coefficients were negative (in this example, Cell D was 11). Thus, judgments based on comparing Cell A with Cell B or Cell C conflicted with judgments based on computing φ or comparing ratios. On the N-CN problems, the absolute numbers in Cell A (e.g., 37) were also greater than the numbers in Cells B (e.g., 15) and C (e.g., 23), but the φ coefficients were positive (e.g., 18 in Cell B). Thus, normative solutions could be based on computing conditional probabilities, comparing ratios, or simply comparing Cell A with Cell B or Cell C.

Participants judged relationship strength on 5-point scales (1 = *strong negative relationship*; 5 = *strong positive relationship*; reversed for two CN and two NC problems). After recoding problems with reversed rating scales, responses were judged normative (scored “1”) when participants indicated that the correlations were negative (i.e., ratings <3) on the CN problems and positive on the N-CN problems (i.e., ratings >3).

### Procedure

The ability measures, because they were timed, were always administered before the other measures. For about half of the participants, the HB battery was presented next, followed by the thinking dispositions questionnaire and the numeracy measure. For the remaining participants, presentation order was the thinking dispositions questionnaire, numeracy test, and HB battery. Order was not significantly related to either normative responses or individual difference variables (largest *r* = 0.11).

## Results

### Conflict and no-conflict problems

To examine whether normative responses were more frequent on N-CN problems than on CN problems, a multivariate analysis of variance, with normative scores on the four tasks as dependent variables and problem type (CN or N-CN) as a within-subjects variable, was conducted. The anticipated main effect of problem type was significant, *F*_(1, 215)_ = 1617.26, *p* < 0.001, η^2^_*p*_ = 0.88: On each task, normative responses were more frequent on N-CN problems than on CN problems, smallest *F*_(1, 215)_ = 295.17, *p* < 0.001, η^2^_*p*_ = 0.60. Mean proportions of normative responses on the conflict and no-conflict problems are presented in Table 1.

**Table 1:** **Mean proportions (and *SD*s) of normative responses on the conflict and no-conflict problems**.

**Task**	**Conflict**	**No conflict**
Base rate	0.49 (0.24)	0.87 (0.21)
Law of large numbers	0.41 (0.23)	0.92 (0.16)
Ratio bias	0.36 (0.21)	0.90 (0.19)
Covariation	0.34 (0.19)	0.88 (0.19)

### Correlations between normative responses and predictors

The next analyses were intended to determine whether no-conflict scores on the four tasks were related to each other, conflict scores, and the individual difference measures (i.e., TD, GA, and numeracy). With the exception of negative correlations between scores on the NC-LLN and CN-COV problems and between scores on the NC-RB and NC-COV problems, no correlations between no-conflict scores on the different tasks or between responses on no-conflict and conflict problems were significant (see Table 2). Similarly, no correlations between the individual difference variables and no-conflict scores were significant (largest *r* = 0.11). Next, the correlations among responses on the conflict versions of the tasks and the correlations among the hypothesized predictors were examined. As expected, and consistent with prior research (Stanovich and West, 1998; Klaczynski, 2001; Chiesi et al., 2011), responses on the conflict versions of each task correlated positively (see Table 2). The predictors were also significantly related (TD-ability = 0.19, *p* < 0.01; TD-numeracy = 0.22, *p* < 0.01; ability-numeracy = 0.31, *p* < 0.001). SAT scores also related to TD, ability, and numeracy (*r*s = 0.27, 0.22, 0.25, respectively, all *p*s < 0.01). However, when they were significant, the relationships between SAT scores and normative responses were weak relative to the correlations between normative responses and the other predictors (see Table 3).

**Table 2:** **Correlations between responses on the conflict and no-conflict problems**.

	**2**	**3**	**4**	**5**	**6**	**7**	**8**
1. CN: BR	0.34^c^	0.34^c^	0.26^c^	−0.02	−0.12	−0.06	0.09
2. CN: LLN		0.36^c^	0.31^c^	0.09	0.11	0.02	−0.03
3. CN: RB			0.40^c^	−0.01	−0.09	−0.01	−0.02
4. CN: COV				0.05	−0.14^a^	0.09	−0.11
5. N-CN: BR					0.03	−0.09	−0.18^b^
6. N-CN: LLN						−0.06	0.02
7. C-CN: RB							0.07
8. N-CN: COV							

a p < 0.05;

b p < 0.01;

c p < 0.001.

**Table 3:** **Correlations between predictors and responses on the conflict problems**.

	**BR**	**LLN**	**RB**	**COV**	**Comp**.
SAT	0.10	0.11	0.15^a^	0.15^a^	0.18^b^
TD	0.27^c^	0.25^c^	0.28^c^	0.32^c^	0.36^c^
Ability	0.28^c^	0.28^c^	0.31^c^	0.30^c^	0.38^c^
Numeracy	0.30^c^	0.25^c^	0.28^b^	0.31^c^	0.39^c^
TD × Ability	0.05	0.09	0.06	0.02	0.08
TD × Numeracy	0.04	0.14^a^	0.12	0.06	0.10
Ability × Numeracy	0.17^b^	0.16^a^	0.19^b^	0.21^b^	0.25^c^
TD × Ability × Numeracy	0.28^c^	0.26^c^	0.26^c^	0.32^c^	0.36^c^

a p < 0.05;

b p < 0.01;

c p < 0.001.

More central to the goals of this investigation were the correlations between conflict responses and the hypothesized predictors. Note that, although the relationships between normative responses and *interactions* between predictors (e.g., Numeracy × Ability) are not typically examined in HB research (see, however, Stanovich and West, 2008; Chiesi et al., 2011; Handley et al., 2011), the study's hypotheses required analyses of these relationships. That is, positive correlations between the TD × Ability × Numeracy interaction and normative responses would be consistent—and thus provide initial support for—the speculation that effects associated with numeracy are constrained by ability and TD.

The correlations of the individual predictors and the predictor interaction terms (computed by standardizing and then multiplying TD, ability, and numeracy scores) to responses on each task and a composite score (normative responses on each task summed and divided by four) are presented in Table 3. TD, ability, and numeracy correlated positively with individual task scores and composite scores, supporting the hypothesis that each variable would predict responses. Of the two-way interactions, the Ability × Numeracy interaction correlated positively with the individual task scores and composite scores. More important, however, were the significant correlations of the TD × Ability × Numeracy interaction to individual task scores and composite scores. As noted above, these particular correlations are consistent with the speculation that the “effects” of numeracy on responses were at least partially constrained by ability and TD. Although promising, the findings from this analysis represent only a first step toward testing the hypothesis. An important second step entailed determining whether the three-way interaction explained variance in normative responses beyond that associated with the individual predictors and the two-way predictor interactions.

### Predicting normative responses

In and of themselves, the correlational findings do not indicate whether TD constrained the numeracy-response relationships or, alternatively, whether TD constrained the ability-response relationships. To reduce the number of additional analyses, subsequent analyses focused on composite scores. This focus is justified by the significant relationships among individual task scores, a principal components factor analysis that yielded a single score with an eigenvalue > 1 (54.18% of the variance among scores; smallest loading = 0.69), and the finding that results for the individual tasks closely paralleled the results from the analyses of the composite^3^.

To determine (a) which predictors accounted for unique variance in normative responses and (b) whether the predictor interaction terms accounted for variance in composite scores beyond the variance associated with the individual predictors, a hierarchical multiple regression analysis was conducted on composite scores. SAT-Math scores were entered at the first step and TD, GA, and numeracy were entered at the second step. To determine whether they accounted for additional variance, the two-way interaction terms were entered at the third step and the three-way interaction term was entered at the final step. Significant contributions of the TD × Numeracy and GA × Numeracy interactions would suggest that numeracy moderated the relationships of TD and GA to normative responses and a significant contribution of the TD × GA × Numeracy interaction would imply that the numeracy-normative response relationship depended on both TD and GA^4^.

Results from the final step, and incremental variance explained by the predictors at each step, are presented in Table 3. In total, the predictors and interaction terms accounted for 35.9% of the variance in composite scores. TD, ability, and numeracy were significant independent predictors, as were the GA × Numeracy and the TD × GA × Numeracy interactions. The significant predictive value of these interactions implies that the effects of ability, numeracy, and TD were less straightforward than implied by the significant beta values of the individual predictors. The three-way interaction, which contributed an additional 2.1% of variance beyond that explained by the other predictors, is particularly important because it implies that the numeracy-normative response relationship depended on GA and TD. Unfortunately, the regression results provide little information regarding the specific nature of the interactive relationships and thus do not fully address the investigation's central hypothesis. Although consistent with the Hypotheses (3a) and (3b), the significant predictive value of the three-way interaction does not indicate that the numeracy-normative association differed for low and high TD participants whose general abilities were low or high and therefore is insufficient evidence for conclusions regarding the constraining effects of TD and GA on the numeracy-normative response association. Consequently, an alternative approach was needed to determine whether the relationship between numeracy and normative responses depended on whether thinking dispositions and general ability were high or low.

### Ability and thinking dispositions as moderators of the numeracy-response association

The hypothesis that the numeracy-response relationship would be significant only if TD and general ability were relatively high is a moderation hypothesis. To test the speculation that numeracy differences depended on both ability and thinking dispositions, Hayes' (2012; for related discussions, see Shrout and Bolger, 2002; Preacher et al., 2007; Hayes, 2013) SPSS macro and, specifically, “process model 3” was used to conduct a “moderated moderation” analysis. In brief, the process macro uses ordinary least squares regression to estimate the coefficients for each predictor and their interactions. Process model 3 is useful in determining the significance of the interactions between and among an independent variable and two moderators. Results indicated whether effects related to numeracy depended on GA and TD and whether the numeracy-composite relationship was significant only when GA and TD were relatively high. As suggested by the foregoing regression analyses, support for the hypothesis was contingent on the significance of the three-way interaction (i.e., Numeracy × GA × TD)^5^.

By default, Hayes' (2012) macro constructs three levels (subsequently referred to as “low,” “moderate,” and “high”; levels are centered around the means; i.e., the mean and ± 1 SD from the mean) for the IV and each moderator. If the three-way interaction is significant, these levels are used to examine the significance of the interaction between numeracy and ability at each level of the moderator (TD). At least in a general sense, the analysis parallels a 3 (numeracy) × 3 (ability) × 3 (TD) analysis of variance. However, unlike analysis of variance approaches, but consistent with current approaches to moderation and mediation (Preacher et al., 2007), bootstrapping procedures are used to obtain 95% confidence intervals. Confidence intervals provided a basis for estimating whether, at each ability level within each TD level, numeracy was significantly related to composite scores. Effects were considered significant when confidence intervals did not contain zero (Hayes, 2012). In the results presented below, LLCI and ULCI refer to lower level and upper level confidence interval, respectively.

To test the hypothesis that numeracy would “directly” affect responses only when TD and GA were high, numeracy was entered as the “independent” variable, TD was entered as a one moderator, and ability was entered a second moderator. SAT-MATH scores and a composite N-CN score were entered as covariates. As in the regression analysis, the covariates, numeracy, GA, TD, and their interactions accounted for 36% of the variance in composite scores, *F*_(9, 206)_ = 12.20, *p* < 0.0001. TD (β = 0.0023, *t* = 2.98, *p* = 0.0032, LLCI/ULCI = 0.0008/0.0038), ability (β = 0.0092, *t* = 3.47, *p* = 0.0006; LLCI/ULCI = 0.0042/0.0152), and numeracy (β = 0.0092, *t* = 2.81, *p* = 0.0054; LLCI/ULCI = 0.0027/0.0156) were significant predictors (neither covariate was a significant predictor, *t*s < 1). The Ability × Numeracy interaction (β = 0.0021, *t* = 2.29, *p* = 0.0178; LLCI/ULCI = 0.0004/0.0038) and the TD × Ability × Numeracy (β = 0.0001, *t* = 2.15, *p* = 0.0322; ULCI/LLCI = 0.0000/0.0002) interactions were significant. The three-way interaction indicated that the effects related to numeracy differed by levels of thinking dispositions and ability ^6^.

**Figure 2:**
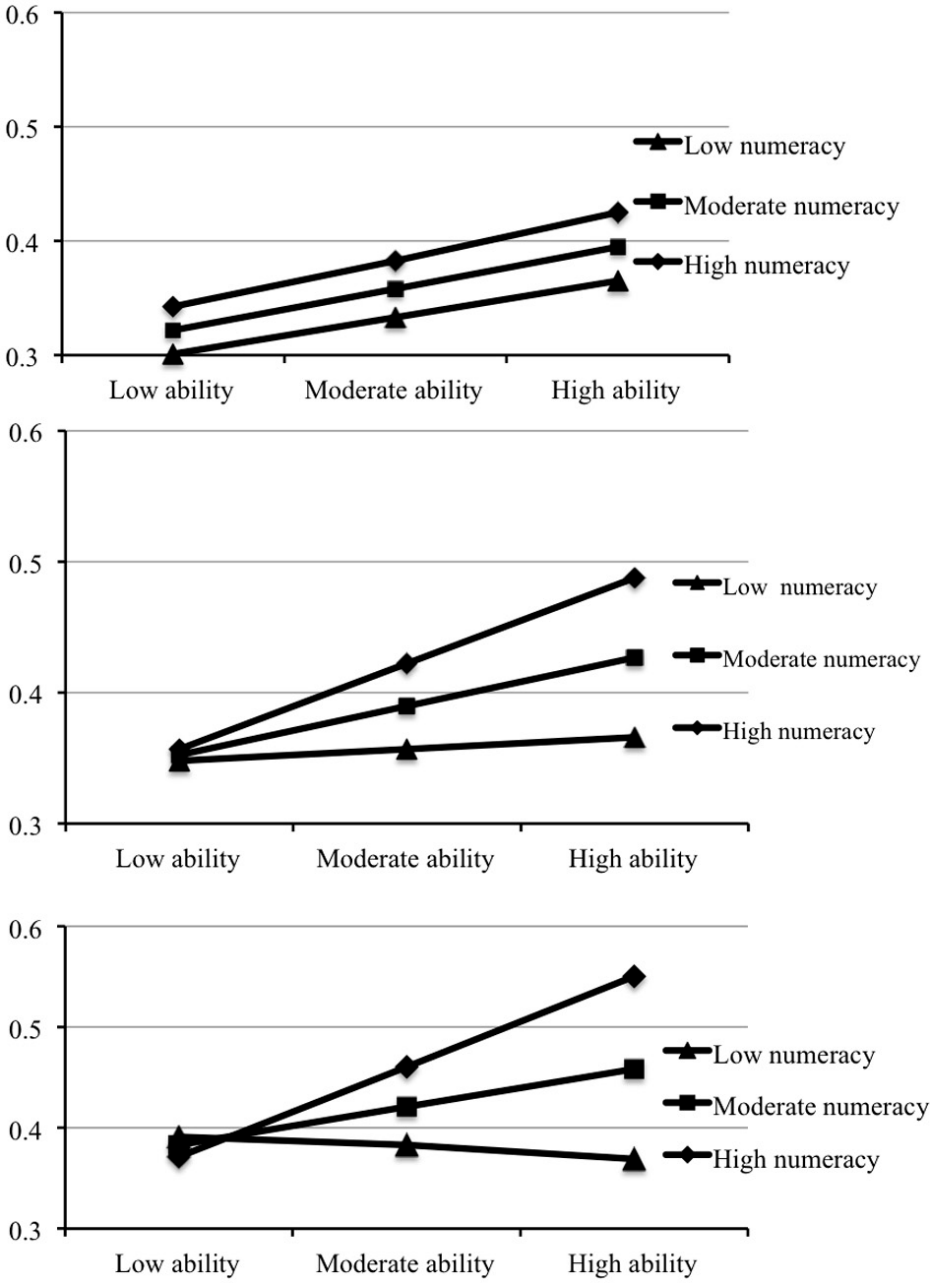
**Moderated moderation results: effects of numeracy levels and ability level on normative responding at low (upper graph), moderate (middle graph), and high (bottom graph) TD levels**.

The results presented in Table 4 show the effects of GA and numeracy at each TD level. As expected, the Numeracy × Ability interaction was not significant at the lowest TD level. Indeed, when TD low, the numeracy-response association was not significant at any ability level. By contrast, at moderate and high levels of TD, the Numeracy × Ability interaction was significant. The additional results shown in the table revealed that, when TD was moderate or high, numeracy directly affected normative responses only if GA was also moderate or high. These findings, depicted in Figure 2, support the general hypothesis that TD and GA constrained the effects of numeracy on responding to probabilistic HB tasks.

**Table 4:** **Hierarchical multiple regression analysis on composite scores (β and *t*-values from final step)**.

**Predictors**	***R*Δ^2^**	***F*Δ**	***B***	**β**	***t***
SAT	0.03	6.14^a^	0.00	−0.02	<1
TD, ability, numeracy	0.27	27.72^c^			
TD			0.00	0.20	3.12^b^
GA			0.01	0.24	3.66^c^
Numeracy			0.01	0.20	3.19^b^
Two-way interactions	0.04	3.66^a^			
TD × Ability			0.00	0.01	<1
TD × Numeracy			0.01	0.05	<1
Ability × Numeracy			0.03	0.18	2.90^b^
Numeracy × Ability × TD	0.02	7.11^c^	0.02	0.17	2.68^b^

a p < 0.05;

b p < 0.01;

c p = 0.001.

**Table 5:** **Moderated mediation results: effects of numeracy on normative responding by TD level and ability level (within TD levels)**.

**Numeracy × ability**	**Predicting composite normative responses**			
	**Estimate**	***t***	**LLCI**	**ULCI**
Low TD	0.0003	<1	−0.00021	0.0028
Ability				
Low	0.0058	>1	−0.10057	0.0174
Moderate	0.0071	1.57	−0.5771a	0.0161
High	0.0085	1.18	−0.1885a	0.0226
Moderate TD	0.0021	2.39^a^	0.0004	0.0038
Ability				
Low	0.0012	<1	−0.0088	0.0113
Moderate	0.0092	2.81^a^	0.0027	0.0156
High	0.0171	4.09^b^	0.0089	0.0254
High TD	0.0038	3.35^b^	0.0016	0.0061
Ability				
Low	−0.0034	<1	−0.0180	0.0113
Moderate	0.0112	2.55^a^	0.0025	0.0199
High	0.0258	5.55^b^	0.0166	0.0350

Note. Numeracy × Ability = Numeracy × Ability interaction at each TD level. Within TD levels, significance of numeracy at low, moderate, high ability levels. Ability and TD levels are derived from means and ± one SD (TD ± 13.85; Ability ± 3.83) from the respective means. LLCI and ULCI = 95% bias corrected lower lever confidence interval and upper level confidence interval, respectively (5000 bootstrap samples).

a p < 0.05;

b p < 0.001.

## Discussion

This study showed that normative responses on no-conflict problems are typically related to neither responses on conflict problems nor thinking dispositions, general ability, or numeracy. By contrast, normative responses on conflict problems related positively to all three individual difference variables. After accounting for variance attributable to thinking dispositions, general ability, and numeracy entered separately, the Thinking Disposition × General Ability × Numeracy interaction accounted for additional variance in normative responses on the conflict problems.

Perhaps the most important contribution of the present research are the findings bearing on hypotheses based on Stanovich's (2009a, 2011) theory of analytic processing. As anticipated by Hypotheses (1) and (2), when TD was low—regardless of whether general ability was low, moderate, or high—and when GA was low—regardless of whether thinking dispositions were low, moderate, or high —numeracy was unrelated to normative responses. Although based on correlational data, these preliminary findings are consistent with the proposed relationship between the reflective and algorithmic levels. Deficiencies at the reflective level appear to limit the efficacy of algorithmic functions. Thus, even the most intellectually able (regardless of numeric ability) solved few probabilistic HB problems correctly when their epistemic beliefs and thinking dispositions were poorly calibrated. Conversely, algorithmic limitations appear to constrain the efficacy of reflective functions: Participants at the highest level of reflective functioning (regardless of numeric ability) performed little better than those at the lowest TD level when they lacked the cognitive resources to conduct reflective operations (e.g., selecting appropriate micro-strategies or mindware, evaluating task representations) and perform correct computations.

Among the most novel contributions of this research, however, were those pertaining to Hypothesis (3b). Consistent with expectations, when TD was moderate-high *and* ability was moderate-high, numeracy associated positively with normative responding. The effects of numeracy were thus moderated by both thinking dispositions and ability. These findings support the position that relatively high levels of reflective and general algorithmic functioning are both necessary for numeracy to influence responding, at least on probabilistic tasks. As indicated in Figure 2, when they lacked *either* the requisite thinking dispositions *or* general intellectual competencies, highly numeric individuals were no more likely than less numeric individuals to respond normatively.

To a greater extent than prior research, these findings support the perspective previously outlined on Stanovich's (2009a, 2011) theory of analytic processing. First, the findings were not limited to a single task but extended across four probabilistic reasoning tasks. Second, few investigations have entailed examinations of the interactive effects of thinking dispositions, general ability, and specific abilities (micro-strategies or mindware) on reasoning. Third, the moderated moderation analytic approach afforded a more precise exploration of the hypothesized relationships than other approaches (e.g., ANOVAs based on median split-created groups). Finally, the results not only implicated numeracy as an important contributor to probabilistic reasoning but also provided theoretically-consistent evidence relevant to the conditions under which numeracy predicts normative responding: When TD *and* GA are both fairly high (note that the precise meaning of “moderate” and “high” TD and GA is relative to the population studied and depends on the measures used to assess these constructs).

The research presented here was concerned with processes that ensue *after* conflict detection and *after* decisions to attempt overriding autonomously-triggered responses with responses based on analytic processing. In the dual-process theory advocated by Evans and Stanovich (e.g., Evans, 2007, 2008, 2012; Stanovich, 2009a,b, 2012; Reyna and Brainerd, 2011; Evans and Stanovich, 2013), rapid processing of problem content activates potential responses. These autonomous responses are not necessarily inadequate or non-normative (Handley et al., 2011; Thompson and Johnson, 2014); instead, they are accompanied by varying “feelings of rightness” (Thompson, 2009; Thompson et al., 2013). Notably, the findings of Handley, Thompson, and colleagues, indicating that normative responses are sometimes automatically activated, provide additional weight to cautionary notes to guard against assuming that analytic processing necessarily underlies normative responses (e.g., Klaczynski, 2001; Reyna et al., 2003; Elqayam and Evans, 2011; Evans, 2011; Reyna and Brainerd, 2011; Stanovich et al., 2011). In the present work, normative responses may sometimes have been activated automatically, a possibility that might partially explain why thinking dispositions, general ability, and numeracy accounted for only 36% of the response variance. As implied below, measures of “feeling of rightness” and inhibition would likely have explained additional variance.

The stronger the “feelings of rightness” elicited by automatic responses, the lower the probability that reasoners will attempt to replace these responses with consciously deliberated answers (Thompson and Morsanyi, 2012; Thompson et al., 2013). The model tested here is therefore likely more relevant to autonomous responses associated with weak “rightness feelings” (or sensing “something fishy” about intuitive responses; De Neys, 2012, p. 31). At a minimum level, the decision to judge the sufficiency of the intuitive responses that trigger weak “feelings of rightness” is a metacognitive, reflective process. However, to further engage analytic processes and fully evaluate automatic responses, both reflective operations and algorithmic resources are required (the latter to compare intuitive responses against responses based on careful deliberation and to internalized standards; see also Moshman, 1998). If an automatically-activated response is deemed inadequate (e.g., inaccurate and/or insufficiently precise), reflective abilities again come into play to assess task requirements, select the appropriate algorithmic skills, and judge the outcomes of implementing those skills. Algorithmic resources are, of course, not only necessary to carry out these procedures and implement specific reasoning, decision making, and computational skills, but also to suppress initial responses and inhibit interference from potentially misleading beliefs activated by task content (e.g., stereotypes) or by the intuitive responses themselves.

To summarize, metacognitive operations at the reflective level determine whether override should be attempted (Klaczynski, 2004; Thompson, 2009; Evans, 2010). Following this decision, the generation of decoupled representations depends on reflective functioning (e.g., recognition of task requirements/structure) which, in turn, is dependent on general algorithmic resources and specific experiences and skills. After such representations are generated, the appropriate mindware (e.g., numeracy)—if available—must be selected (Stanovich, 2012). Even if available, correct strategy/skill selection does not guarantee that implementation will be effective. Inability to sustain generated representations and inhibit autonomous responses (effortful processes requiring both algorithmic resources and reflective dispositions; see Stanovich and West, 2008) can lead to interference from non-essential task contents and implementation errors (see also the discussion of “levels of rationality” in Reyna et al., 2003). Clearly, as anticipated by the arguments and supported by the evidence proffered by Reyna et al. (2003) and others (e.g., Evans, 2011; Stanovich et al., 2011; Klaczynski, 2013), attempts to override responses based on autonomous processing are neither invariably successful nor invariably lead to normative responses.

By themselves, neither algorithmic capabilities (including specific mindware) nor competence at the reflective level sufficed to produce normative responses. In Stanovich's theory, the reflective-algorithmic relationship is reciprocal because reflective operations are necessarily constrained by available resources (Stanovich and West, 2008). Thus, even those at the highest general ability and numeracy levels typically gave non-normative responses when their reflective dispositions and skills were poor (see also Overton, 1990; Amsel et al., 2008; Chiesi et al., 2011; Ricco and Overton, 2011; Morsanyi and Handley, 2013). Several reflective-level difficulties, such as failures to accurately assess task requirements, attend to numerical information in accurate representations, select appropriate computational skills, monitor numeric functions and outputs, or equate *subjectively-adequate* responses with normative responses, could have led to non-normative responses. Conversely, even participants at the highest levels of reflectivity and numeracy typically gave non-normative responses if their general ability scores were low. Lacking the requisite resources to implement and monitor their numeric skills while maintaining decoupled representations (see Stanovich et al., 2011, 2012; Stanovich, 2012), these individuals performed no better than those at low levels of reflective functioning and numeracy.

The findings support the theory of analytic processing proposed by Stanovich (2009a, 2011) and implicate numeracy as a specific algorithmic skill likely to further our understanding of the processes underlying performance on HB tasks. Research on the role of instructions in reasoning is also consistent, and can be interpreted from the perspective of, Stanovich's theory. Evidence from several reports indicates that reliance on heuristics decreases and normative responses increase when participants are instructed to think logically (e.g., Denes-Raj and Epstein, 1994). Recent findings (e.g., Macpherson and Stanovich, 2007; Evans et al., 2010; Handley et al., 2011; Morsanyi and Handley, 2012; Morsanyi et al., 2012) have further demonstrated that such instructions improve responding primarily among high ability participants and that, in the absence of such instructions, general ability is unrelated to responding on some tasks. If conceived as externally-imposed surrogates for well-calibrated thinking dispositions—or as cues to engage in reflective-level operations—logic instructions should only benefit those with sufficient algorithmic capacity to not only keep the instructions in mind but also construct accurate representations and conduct the relevant computations. Just as it constrains reflective-level functioning, general ability limits the efficacy of logic instructions.

Despite evidence consistent with the view that a function of the reflective level is to select, guide, and monitor algorithmic operations and that algorithmic limitations constrain not only these reflective operations but also the implementation of specific abilities, there are reasons to guard against interpreting the current findings as definitive support for this theoretic position. Specifically, the correlational nature of the study prohibits the conclusions that thinking dispositions *constrained* the functioning of general ability and that limitations in general ability *constrained* numeric operations (see Footnotes 1 and 6). For instance, the hypothesized relationship between thinking dispositions and general ability is reciprocal; however, it was not possible to examine directly bidirectional (or unidirectional) causal relationships in the present work. Even if the causal relationships operate as hypothesized on probabilistic tasks, the model does not explain findings that, on some HB tasks, (a) thinking dispositions sometimes predict performance but general ability does not, (b) general ability sometimes predicts performance but thinking dispositions do not, and (c) neither thinking dispositions nor general ability relate positively to performance (e.g., Klaczynski, 2000; Stanovich and West, 2008; Thompson and Johnson, 2014). These mixed and sometimes null findings may, to some extent, be attributable to the fact that measures of general ability and thinking dispositions are imperfect indexes of algorithmic and reflective functioning. Replications of, for instance, research on myside biases that utilizes more specific (and/or more extensive) measures of algorithmic (e.g., inhibition) and reflective (e.g., metacognitive monitoring) processes would likely contribute valuable insights toward explaining these findings.

Another issue is that the individual differences measures accounted for only 36% of response variance. One reason for this, alluded to earlier, is that normative responses are sometimes activated automatically. In such instances, complete engagement of analytic resources is not always necessary (reasoners may even forgo checks of response override when automatic normative responses are accompanied by strong feelings of rightness). An expansion of this account may also help explain the unexplained variance: When initial responses prompt attempts to override and to construct decoupled representations, it is conceivable that the process of assessing task requirements automatically activates normative responses. That is, the effort that goes into override and/or decoupling may be sufficient to trigger normative responses. In such cases, algorithmic resources would be taxed little (see Thompson and Johnson, 2014) and reflective operations would be relatively limited (e.g., monitoring computation quality would neither be necessary nor possible). This account, however, awaits empirical testing.

Nonetheless, at least on probabilistic reasoning tasks, the combination of well-calibrated beliefs and intellectual dispositions with moderate-high cognitive ability may well lead to normative responses *if* specific micro-strategies or mindware (e.g., numeracy) are available. The findings thus lend additional substance to recent discussions of dual-process theories, support the distinction between the reflective and algorithmic levels of analytic processing, and contribute new data to the growing literature on numeracy. Even so, additional research examining the interactions among thinking dispositions, general ability, and specific abilities is clearly needed. In conducting these investigations, theory-driven moderation (and mediation) analyses will likely yield results more informative than those based on less precise analyses (e.g., ANOVA). When coupled with findings from experimental research, our understanding of the processes that underlie judgments, reasoning, and decisions will likely improve considerably. Arguments over whether responses judged normative should be considered prescriptive can be better addressed empirically. As an example, if general abilities are subordinated to thinking dispositions/epistemic regulation and the latter can be acquired through formal and informal tuition—and if some specific algorithmic abilities are educable—then the possibility the reducing the gap between traditional norms (“what ought”) and actual behavior (“what is”) remains open (for discussion and alternative perspectives, see Elqayam and Evans, 2011).

### Conflict of interest statement

The author declares that the research was conducted in the absence of any commercial or financial relationships that could be construed as a potential conflict of interest.

## Footnotes

^1^The theoretical speculations advanced here imply causal relationships among thinking dispositions, general ability, and numeracy. However, with the exception of the conflict/no-conflict problem comparisons, the research was correlational. In the Results, terms that connote causality are sometimes used because of space considerations and because such terms (e.g., “direct” effects) are used in discussions of moderation. Although the observed relationships may be causal, they should be interpreted cautiously and with consideration of alternative explanations (see Discussion).

^2^In a larger investigation, numeracy was only related weakly to responses on non-probabilistic problems. Despite GA and TD correlations to responses similar to those reported here, the TD × GA × Numeracy interaction was not significant; instead, the TD × GA interaction was a significant predictor of responses.

^3^In subsequent analyses of composite scores, similar results obtained when factor scores were analyzed.

^4^No interaction that included total SAT scores or SAT-MATH scores related to, or predicted, composite scores. However, because they related to composite scores, SAT scores were included in subsequent analyses as covariates.

^5^Similar results obtained from a 2 (TD group) × 2 (ability group) × 2 (numeracy group) ANOVA on composite scores. In the low TD group, no effects related to numeracy were significant (*p*s > 0.20). In the high TD group, the Ability × Numeracy interaction was significant, *F*_(1, 103)_ = 16.71, *p* < 0.001, η^2^_*p*_ = 0.07. When TD was high, but ability was low, scores did not differ in the by numeracy group, *F* < 1. However, when TD and ability were high, the high numeracy group performed better than the low numeracy group, *F*_(1, 61)_ = 35.83, *p* < 0.001, η^2^_*p*_ = 0.37. Similar results obtained when Bayes factor—indicating the likelihood that the high and low numeracy groups differed—was computed at each TD and GA level (with *r* was set at 0.50; see http://pcl.missouri.edu/bayesfactor). Comparisons between the high and low numeracy groups for (a) low TD/low GA participants, (b) low TD/high GA participants, (c) high TD/low GA participants, and (d) high TD/high GA participant yielded Bayes factors of 1.113, 0.417, 1.923, and 7.283, respectively (the final factor is considered moderate/strong).

^6^Hayes (2012) refers to Process 3 as a “moderated moderation” analysis, intended to determine whether the effects of an independent variable interact with the effects of two other variables (moderators). Although the decision to enter TD and GA as moderators and numeracy as the IV was theoretical, the analysis is nonetheless analogous to an analysis of variance (see Footnote 5) with three levels for each “IV.” As such, the three-way interaction was significant regardless of which variables were entered as moderators and which was entered as the IV. For instance, with numeracy was the IV, GA as the first moderator, and TD as the second moderator, the variance explained was identical. The primary difference is that, instead of presenting the GA × Numeracy interaction (and simple effects of numeracy) within each TD level, this alternative analysis indicated whether the TD × Numeracy interaction was significant at each GA level and, within GA levels, the direct affects of numeracy when TD was low, moderate, and high. However, in contrast to the findings presented here, the TD × Numeracy interaction was significant only when GA was high. Otherwise, the results of the follow-up analyses were analogous to those in Table 5: Numeracy directly affected responses when GA was moderate and high and when TD was also moderate and high.
